# Outreach and Support in South London for Bipolar At-Risk States: pilot implementation of a specialist preventive service for adolescents and young adults at risk of developing bipolar disorders

**DOI:** 10.3389/fpsyt.2026.1773189

**Published:** 2026-08-28

**Authors:** Antonio Melillo, Andrea De Micheli, Sunil Nandha, Romayne Gadelrab, Sameer Jauhar, Allan Young, Matilda Azis, Mirko Manchia, Andrea Pfennig, Armida Mucci, Silvana Galderisi, Paolo Fusar-Poli

**Affiliations:** 1 Department of Mental and Physical Health and Preventive Medicine, University of Campania “L.Vanvitelli”, Napoli, Italy; 2 Early Psychosis: Interventions and Clinical-Detection (EPIC) Lab, Department of Psychosis Studies, Institute of Psychiatry, Psychology & Neuroscience, King’s College London, London, United Kingdom; 3 Outreach and Support in South London (OASIS) Service, South London and Maudsley National Health Service Foundation Trust, London, United Kingdom; 4 Department of Psychological Medicine, Institute of Psychiatry, Psychology & Neuroscience, King’s College London, London, United Kingdom; 5 Division of Psychiatry, Department of Brain Sciences, Imperial College London, London, United Kingdom; 6 Section of Psychiatry, Department of Medical Sciences and Public Health, University of Cagliari, Cagliari, Italy; 7 Department of Pharmacology, Dalhousie University, Halifax, NS, Canada; 8 Department of Psychiatry and Psychotherapy, Carl Gustav Carus University Hospital, Medical Faculty, Technische Universität Dresden, Dresden, Germany; 9 Department of Brain and Behavioral Sciences, University of Pavia, Pavia, Italy

**Keywords:** affective disorders, bipolar risk, early intervention, prevention, semistructured interview for bipolar at risk states, SIBARS, young people

## Abstract

Bipolar disorders (BD) are associated with high disability, comorbidities, and premature mortality, yet diagnosis is often delayed by several years. Research has identified bipolar at-risk states (BARS), but no study has tested the feasibility and acceptability of a dedicated specialist service. Consequently, evidence is lacking on optimal service set up, outreach strategies, and referral pathways required to effectively implement a BD prevention service. Outreach and Support in South London (OASIS)-BAR was piloted for 18 months as the first National Health Service (NHS)-funded standalone specialist service for individuals at risk of BD. The service, co-designed with experts by experience, operated in Lambeth, London, a socially deprived and ethnically diverse borough. Feasibility was evaluated through service set up, efficiency (outreach performance, waiting times, detection rates, treatment provision), and continuity of care (successful discharge planning). Acceptability was assessed through service engagement and qualitative feedback from service users and stakeholders. Of 28 referrals, 17 were assessed and 10 (58, 8%) met SIBARS criteria. The mean waiting time from referral to complete assessment was 22.4 days. All service users received medical reviews (100%), while most received psychotherapy (66, 7%) and occupational support (77, 8%). Engagement was satisfactory (mean length = 271 days). Service users expressed high satisfaction, and professional stakeholders emphasised the service’s role in addressing unmet needs and alleviating caseload burden on other services. OASIS-BAR pilot implementation demonstrated the feasibility and acceptability of a standalone service for BD prevention within the NHS. Findings support the development of similar innovative services, which may strengthen early detection and preventive care, ultimately improving long-term outcomes for many young people.

## 1 Introduction

Indicated prevention of serious mental disorders has become a prominent area of research and clinical care in psychiatry (1). The most established indicated prevention model in clinical psychiatry is the Clinical High Risk state for Psychosis (CHR-P), which led to the implementation of specialised services across the world (2, 3) and has impacted national (4) and international (5) clinical guidelines as well as diagnostic manuals (6). Following the CHR-P model, researchers have developed constructs identifying risk states for other mental disorders, such as major depressive and bipolar disorders (1, 7–9).

Bipolar disorders (BD) are highly prevalent conditions, with a global estimate of 489.8 cases per 100, 000 people (10). They are characterised by a high disability burden as well as several comorbidities, which contribute, together with the increased risk of suicide, to a reduction in life expectancy of 12 to 14 years (11). BD typically manifest in adolescence or early adulthood and may exhibit a progressive trajectory for a significant proportion of individuals, marked by increasingly frequent relapses, decreased response to treatment and cognitive impairment (12–15). Research shows there is an average 9-year delay between the first onset of symptoms and appropriate diagnosis and treatment (11, 12). This delay is particularly concerning, as evidence shows it is associated with inappropriate treatment, treatment resistance, more frequent relapses and higher rates of physical comorbidities (12, 16). In addition, a BD diagnosis is often preceded by years of other mental health issues (e.g., anxiety disorders), subclinical affective symptoms, substance use and help-seeking behaviour (17–19). Despite this growing evidence highlighting the need to improve early detection and care of subjects at risk of developing BD, individuals often remain undetected and do not receive specialised support.

Although approximately 16% of CHR-P individuals have been found to be at risk of developing BD (20), existing CHR-P assessment tools, such as the Comprehensive Assessment of At-Risk Mental State (CAARMS) (2), cannot predict the emergence of BD (20–22). To address this gap, the first step has been to develop and validate new operational criteria defining bipolar at-risk states (BARS) (7, 8, 23–26). The subsequent step has been to develop and validate new psychometric assessment tools, such as the Structured Interview for Bipolar At Risk State (SIBARS) (9), designed to complement CHR-P assessment tools to identify BARS. The SIBARS has shown excellent internal consistency and inter-rater reliability, moderate-to-strong convergent validity with existing clinical assessment tools, and good prognostic accuracy in longitudinal studies (9, 27). Finally, a specific and remotely delivered SIBARS training package has also been set up and is available to healthcare professionals or clinical researchers worldwide (see the “SIBARS Practitioner Training Course”, available at https://maudsleylearning.com/courses/structured-interview-for-bipolar-at-risk-states-sibars/).

Despite the growing literature on BARS and the increasing availability of validated assessment tools, evidence on the feasibility and acceptability of dedicated services for the prevention of BD remains limited. While several initiatives have adopted transdiagnostic models of preventive care (28–30), no study to date has tested the feasibility of implementing a service dedicated to the care of individuals at risk for BD. Furthermore, the application of the SIBARS has never been evaluated in this context of the NHS. For this reason, no evidence currently exists on the optimal configuration and staffing composition of a dedicated service, on the role of experts by experience (EbE) in its design, or on the outreach, governance, and referral strategies required for its integration within existing early intervention networks. Consequently, key feasibility and acceptability parameters remain unexplored, including outreach capacity, service efficiency and coordination with other services to ensure continuity of care. Similarly, its acceptability for both service users and mental health professionals has yet to be evaluated.

For over two decades, the Outreach and Support in South London (OASIS) service has been a pioneering model for the early detection and treatment of CHR-P individuals and provides a successful reference model for multidisciplinary and transitional indicated prevention services (31, 32). Building on this expertise, and with the aim to address these knowledge gaps, the service has been piloting the first NHS-funded standalone service dedicated to preventing the development of BD in individuals at risk (OASIS-BAR). The new service was co-designed with EbE and aimed at enhancing the preventive capacity of the current OASIS service network (hereafter referred to as OASIS-CHR-P), fostering earlier detection, timely intervention, and the production of novel clinical and research insights into BARS.

The aim of the present contribution is to test for the first time the feasibility and acceptability of implementing preventive care for BD in the NHS in a socially deprived inner-city area with a large ethnic minority population and a high incidence of severe mental disorders.

## 2 Methods

### 2.1 Duration

The OASIS-BAR project started in March 2023 and was designed as a two-year implementation programme. Due to a sudden withdrawal of funding because of financial NHS constraints (33), which led to the premature discontinuation of care provision, the project lasted 18 months. The first seven months focused on service set-up, staff recruitment and training; the subsequent six months on outreach, clinical assessment, and delivery of care; the final 5 months were particularly dedicated to discharge planning of service users to appropriate services.

### 2.2 Feasibility

Feasibility was assessed based on three performance indicators:

- Service set up: staff recruitment and training
- Service efficiency: outreach and referral pathways performance (i.e. number of referrals); waiting times from referral to assessment; SIBARS detection rates; treatment provision (i.e., number of provided sessions with psychologist, psychiatrist, key workers/occupational therapist)
- Continuity of care: successful referral of patients to other NHS services due to the termination of the project

### 2.3 Acceptability

Acceptability was assessed through service-user engagement (length of stay) as well as through a case audit gathering feedback from both service users and professionals across South London NHS Foundation Trust (SLaM)who had interacted with the OASIS-BAR service. All service users and professional stakeholders who had interacted with the service were invited to provide feedback on a voluntary basis, and all collected feedback is reported.

### 2.4 Service characteristics

#### 2.4.1 Catchment area

The OASIS-BAR project was implemented in the context of the SLaM. SLaM is Europe’s largest mental health provider, serving a population of 1, 342, 303 (population aged 14–35: 492, 393, 36.7%) (34). SLaM provides OASIS-CHR-P services across four South London boroughs (Lambeth, Southwark, Croydon and Lewisham) (31). The OASIS-BAR project was piloted in Lambeth. The borough has a total population of 316, 920 (population aged 14–35: 136, 640, 43.1%) (34). The borough is ethnically diverse, with 55% of residents self-identifying as White, 24% as Black (primarily African and Caribbean), 8% as Mixed, and 7% as Asian (35). Notably, 38.6% of the population were born outside the UK (34, 35). According to the 2021 Census, nearly half of the borough’s households were classified as deprived in at least one of the four dimensions of social deprivation (employment, education, health and disability, housing) (35). Presumably due to the accumulation of these and other risk factors (36), the incidence of serious mental disorders in Southeast London is one of the highest in the world (37, 38). The incidence of psychotic disorders in Lambeth has been estimated to be 71.9 cases per 100, 000 person-years (31, 39). In relation to BD, no recent borough-level data is currently available, but historical estimates for Southeast London reported an incidence over twice as high as other English cities (40). In England, the 2021 Global Burden of Disease Study (41) estimated the incidence of BD at 89 cases per 100, 000, slightly above the global incidence (86 cases per 100, 000).

#### 2.4.2 Service configuration

OASIS-BAR was an NHS-funded transitional (i.e. from age 14 to 35) and specialist mental health service for help-seeking individuals at risk of developing BD. The service was co-designed with EbE and a multidisciplinary panel of professionals, including psychologists, psychiatrists, keyworkers, specialist registrars. EbE informed the configuration of the service by contributing to the design of pathways, care processes, and communication strategies.

Inclusion criteria at OASIS-BAR were: (i) being aged 14-35, (ii) having the general practitioner (GP) in the borough of Lambeth, (iii) being help-seeking, and (iv) meeting SIBARS criteria (9, 27) (see section 2.4.5 for details on the SIBARS).

The core aims of the OASIS-BAR were:

- To detect SIBARS criteria in young people and reduce their disability
- To prevent the onset of a first episode of BD in people meeting SIBARS criteria
- To improve BD outcomes by reducing the duration of untreated disorder

Following the OASIS-CHR-P model, OASIS-BAR operated within the broader early intervention pathway of the SLaM, complementing the existing early intervention services for first-episode mood and psychotic disorders. These include, in addition to the OASIS services, the Lambeth Early Onset (LEO), the Southwark Treatment for Early Psychosis (STEP), the Lewisham Early Intervention Service (LEIS), the Croydon Outreach and Assertive Support Team (COAST), and the Lambeth Hospital inpatient units. OASIS-BAR was physically co-located within OASIS Lambeth. In addition, SLaM has recently implemented a single point of access (SPA), designed to facilitate access to appropriate care through a youth-friendly and soft entry point, aligning with other established service models (42–44). SLaM’s SPA includes a team of mental health professionals, social workers, and support workers who can visit young individuals who seek mental health support within 24 hours and refer them to the appropriate SLaM team, including OASIS and OASIS-BAR. Like OASIS-CHR-P services, OASIS-BAR collaborated closely with child and adolescent mental health services (CAMHS) as well as with local primary care and community mental health services (CMHS), ensuring smooth pathways to care and facilitating referrals.

#### 2.4.3 Outreach strategies

OASIS-BAR conducted a comprehensive outreach programme between months 7 and 13, with the goal of raising awareness on BD prevention, promoting referrals and informing/educating other professionals. The outreach campaign included regular promotional presentations and community visits, building on the established OASIS-CHR-P programme and its collaborations with local organisations. Importantly, the outreach activities were also informed by the contribution of experts by experience, helping refine the messaging and engagement strategies. The OASIS-CHR-P outreach programme has been more comprehensively described in previous papers (31, 32, 45).

#### 2.4.4 Psychometric assessments

The SIBARS (9, 27) is a semi-structured instrument developed to complement the assessment of psychosis risks through CAARMS and to detect BARS by assessing subthreshold affective symptoms, temperamental traits, and family risk factors. Family risk factors are assessed within the SIBARS interview, primarily based on service-user report and corroborated where available. The scale delivers three mutually exclusive outcomes: a) BD or related disorders are already present; b) a bipolar at-risk state (BARS); c) not at risk. Among BARS cases, the SIBARS further distinguishes three subgroups reflecting distinct vulnerability profiles: Group 1, characterised by attenuated mania (with or without mixed features); Group 2, depression with mixed features or cyclothymic temperament; and Group 3, first-degree family history of BD coupled with depression or cyclothymic temperament (9). In the original validation (9), refining the clinically-rated BAR criteria into the operationalised BARS criteria improved prognostic accuracy for incident bipolar disorder from poor (Harrell’s C = 0.66) to adequate (Harrell’s C = 0.78; HR = 12.36 for BARS-positive vs BARS-negative individuals), with high inter-rater reliability (weighted κ = 0.86); the SIBARS interview replicated this adequate accuracy in an independent prospective CHR-P cohort (Harrell’s C = 0.74). Subsequent work has further characterised its internal consistency and convergent validity (27). A detailed description of the SIBARS, including its administration, scoring procedures, SIBARS subgroups, psychometric properties, and prognostic validity, is available in the original methodological and validation papers (9, 27).

In addition to the SIBARS and CAARMS (9, 27), OASIS-BAR offered a range of psychometric assessments, both self-report clinical scales and diagnostic interviews of other mental health conditions (e.g., ADHD) according to individual needs.

#### 2.4.5 Offered preventive treatments

The OASIS-BAR service was designed to offer a personalised package of preventive services, adapted from the established OASIS multidisciplinary model and tailored to BAR-specific clinical needs through co-design with experts by experience. Core components included:

- Psychological therapy (CBT-based but also integrating elements from systemic and family approaches)
- Medical reviews
- Structured keyworker support addressing emotional wellbeing, social inclusion, and practical needs such as education, employment, housing, and finances.
- Additional *ad-hoc* support (family intervention work, carer support and other psychosocial interventions, pharmacological therapy when clinically indicated)
- Lifestyle management
- Psychoeducation on modifiable risk factors for BD
- Continuous monitoring of individual vulnerability through multidisciplinary case discussion.
- Prompt referral to local early intervention teams in case of first affective episode

When under the age of 18, service users remained under the care of CAMHS while receiving additional OASIS-BAR interventions. The services provided by OASIS-BAR were designed to be centred on the individual’s goals, values and needs, to empower young people to manage mood difficulties and to promote resilience. Consistently with the established OASIS model (31), psychological therapy consisted of CBT for emerging mental disorders delivered flexibly according to presenting needs and under regular supervision; occupational therapy was similarly provided as part of this needs-based care rather than as a stand-alone manualised intervention.

## 3 Results

### 3.1 Feasibility

#### 3.1.1 Service set-up

The initial staff recruitment plan consisted of a multidisciplinary team comprising a full-time team leader, senior psychologist, psychologist, assistant psychologist, two keyworkers, one part-time consultant psychiatrist, one honorary consultant psychiatrist and a part-time specialist registrar. The staff were recruited to work across adult and children and adolescent directorates, in line with the transitional nature of the OASIS-BAR.

However, the service did not receive the planned funding required to implement the initial recruitment plan. The final service team therefore included a team leader [Band 7; NHS Agenda for Change pay bands (46)], senior psychologist (Band 8a), a part-time consultant psychiatrist (0.5 whole-time equivalents, WTE) and an honorary consultant psychiatrist (0.4 WTE), at an indicative annual staffing cost of approximately £162, 680 (46, 47). For comparison, the OASIS team in Lambeth and Southwark includes a full-time team leader, two part-time consultant psychiatrists, a part-time trainee in psychiatry, a full-time assistant psychologist, a full-time clinical psychologist, a part-time clinical psychologist and two full-time allied mental health professionals.

Staff training performance was satisfactory: all team members were trained in the SIBARS (v2.1/2020) and the CAARMS, received CBT training and supervision from senior researchers, and were trained to work within a preventive clinic according to multidisciplinary team frameworks; all remained compliant with Trust-mandated training requirements. Training was delivered in collaboration with the established OASIS services, supporting transfer of expertise from a long-standing preventive programme (31); the integration of clinical care and research provided a further training route, as participation in research studies routinely entails study-specific (*ad hoc*) clinician training.

#### 3.1.2 Service efficiency

##### 3.1.2.1 Outreach and referral pathways performance

**Figure 1:**
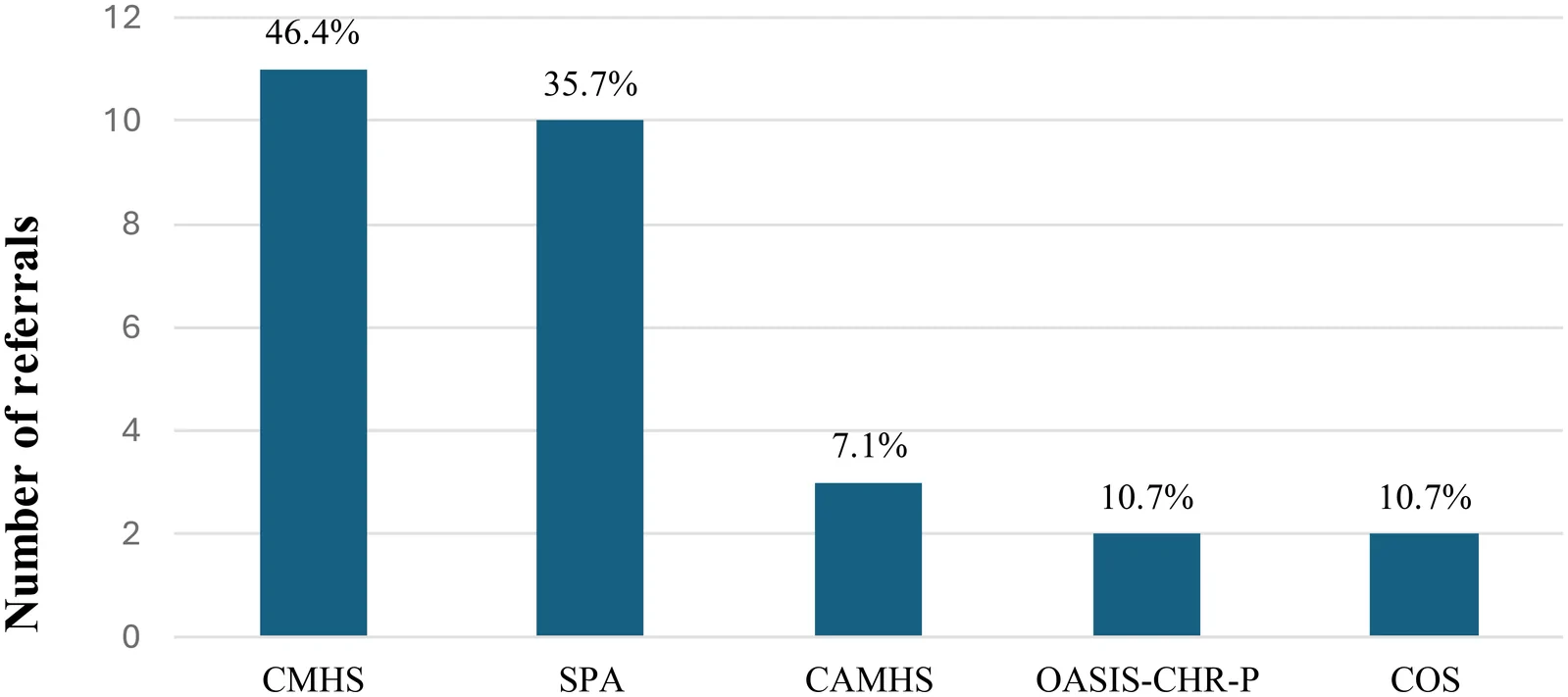
Referral sources. CAMHS, Child&adolescent mental health services; CMHS, community mental health services; COS, crisis outreach services; OASIS-CHR-P, Outreach and Support in South London service for CHR-P individuals; SPA, single point of access.

A total of 28 referrals were made to OASIS-BAR during the six-month period dedicated to outreach, clinical assessments, and delivery of care (months 7–13). See **Figure 1** for the referral sources and **Table 1** for the socio-demographic features of referred individuals.

**Table 1:** Baseline characteristics of the referred individuals (N = 28).

Features	Overall sample (N = 28)
Mean age (SD)	25.8 (6.27)
Gender (n. of females)	17 (60.7%)
Self-reported ethnicity* (%)	
British – White	11 (42.3%)
British – Black	2 (7.7%)
British – Asian	1 (3.8%)
Mixed ethnicity	2 (7.7%)
Asian	3 (11.5%)
White – Other	7 (26.9%)
Employment status**	
Full-time employed	15 (60%)
Part-time employed	2 (8%)
Student	5 (20%)
Unemployed	3 (12%)
Housing status**	
Lives with family	10 (40%)
Lives with partner	6 (24%)
Lives with flatmates	2 (8%)
Lives alone	7 (28%)

*Not reported, n=2; **Not reported, n=3; percentages are calculated on cases with available data.

##### 3.1.2.2 Waiting times from referrals to completed assessment

OASIS-BAR demonstrated efficiency in terms of timely response to referrals, with a mean waiting time from referral to completed assessment of 22.4 days (SD = 19.9).

##### 3.1.2.3 SIBARS detection rates

Of the 28 individuals who were referred to OASIS-BAR, 17 were assessed for CHR-P and SIBARS criteria, while 11 individuals were not assessed, either because they were not engaging with the service (n = 3) or because following consultation and liaison with the referral sources, they were redirected to more appropriate services (CMHS, n = 5; CAMHS, n = 1). Finally, 2 referrals were rejected due to service interruption.

Of 17 assessed individuals, 10 (58.8%) met SIBARS criteria and three met criteria for a BD (overall detection rate = 76.5%). Of the 10 individuals meeting SIBARS criteria, three met criteria for attenuated mania (Group 1), two for depression with mixed features or cyclothymic temperament (Group 2), one for genetic risk with depression or cyclothymic temperament (Group 3), and four met criteria for more than one group (attenuated mania + genetic risk with depression or cyclothymic temperament = 2; attenuated mania + depression with mixed features or cyclothymic temperament = 2). Importantly, two of the 10 individuals meeting SIBARS criteria also met CHR-P criteria. As such, individuals meeting SIBARS criteria were offered preventive care (see **Table 2** for the baseline characteristics of individuals meeting SIBARS criteria), while the remaining seven individuals were excluded, either due to receiving a diagnosis of a serious mental disorder (BD type 1, n=2; BD type 2, n=1; first-episode psychosis, n=1), not completing the assessment (n=2) or residing outside of Lambeth (n=1). All excluded individuals were redirected to more appropriate services following consultation and liaison with both GP and referral sources (Lambeth LEO, n=2; CMHS, n=4; Lambeth SPA, n=1). See **Table 1** for baseline features of the SIBARS sample (n=10).

**Table 2:** Baseline characteristics of individuals meeting SIBARS criteria (N = 10)*.

Features	SIBARS sample (N = 10)
Mean age (SD)	24.43 (5.59)
Gender (n. of females)	5
SIBARS subgroups	
Group 1	3
Group 2	2
Group 3	1
Criteria met for more than one group	4

*Given the small sample size, we do not report data on ethnicity, housing and employment status due to privacy concerns. SIBARS Group 1: Attenuated mania; SIBARS Group 2: Depression with mixed features or cyclothymic temperament; SIBARS Group 3: Genetic risk with depression or cyclothymic temperament. .

##### 3.1.2.4 Treatment provision

Of the 10 individuals who were offered access to the OASIS-BAR service, one did not engage. All remaining service users (n=9) received medical reviews (mean=8.4, SD = 4.43). Seven received occupational therapy sessions (mean=8.2, SD = 3.77), and six received CBT-based psychotherapy sessions with a psychologist (mean=21.7, SD = 9.7). Six individuals received pharmacological treatment (antidepressant: n=3; mood stabilizer: n=1; antidepressant + mood stabilizer: n=2).

##### 3.1.2.5 Continuity of care

**Figure 2:**
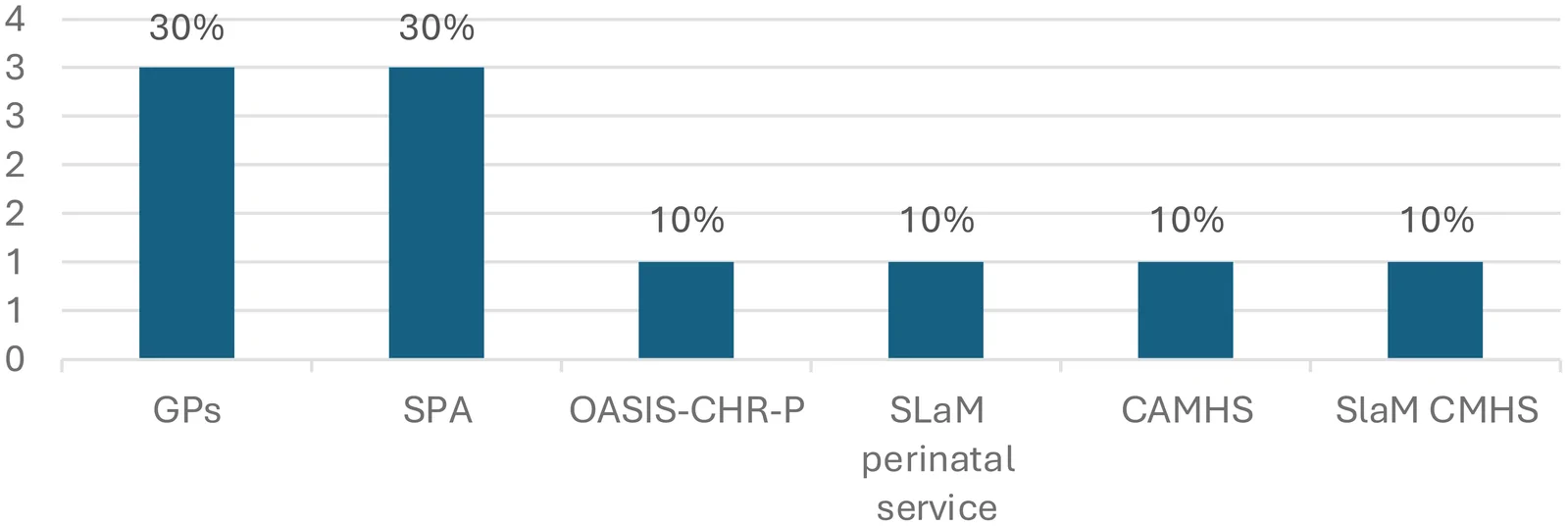
Discharge destinations. CAMHS, Child&Adolescent Mental Health Services; CMHS, Community Mental Health Services; GP, General Practitioner; OASIS-CHR-P, Outreach and Support in South London service for CHR-P individuals; SLaM, South London and Maudsley NHS Foundation Trust; SPA, Single Point of Access.

Following the termination of the programme, all service users were successfully redirected to appropriate services after evaluation of their needs and liaison with their GPs. See **Figure 2** for discharge destinations of service users.

### 3.2 Acceptability

#### 3.2.1 Engagement with services

Engagement with services was satisfactory (mean length of stay=271 days, SD = 127.24, 1 early discontinuation).

#### 3.2.2 Qualitative feedback

Qualitative feedback from professional stakeholders consistently emphasised the clinical value and usefulness of OASIS-BAR as a valuable additional resource for pre-existing SLaM early intervention services, both to help reduce the caseload burden of other services and to meet the need for specialised care for individuals meeting criteria for BARS. A clinical expert who had been working at Lambeth’s SPA during the implementation of OASIS-BAR shared, *“I am convinced of the need of OASIS-BAR. I think we often found people with a bipolar affective disorder diagnosis query and would send them to STS [Short-term support services]. I think [OASIS-BAR services] are valuable and help prevent the over-loading of CMHTs that are already struggling.”* Similarly, a social worker underlined, “*I think it would be a very useful resource to have.”* Another professional stressed that “*this is a service that has been necessary for what we have been clinically observing for years: groups of young people with bipolar spectrum disorders and comorbidities, having utilised services for years and not having received adequate care.”* Feedback from service users highlighted positive experiences both with the provided services and with the SIBARS framework, which provided them an explanation for their difficulties. One patient, for instance, highlighted that *“I have found this more useful than other institutions (e.g., CAMHS) because it helped me understand the difficulties I’ve had with mood, attention, how that impacts my life in positive and negative ways, stress management, and how to cope with negative external situations by holding myself to a routine and unlearning negative thought patterns.”* Consistently with these comments, stakeholders expressed negative feedback regarding the premature interruption of the service. The abrupt ending of the service led to staff dissatisfaction, as they were unable to fully fulfil their roles and deliver the level of care they had aimed for. This affected team morale and overall job satisfaction. As one team member noted, *“It was difficult to explain to patients that the services they had been offered were going to be interrupted earlier than expected.”* The closure of the service also impacted referral partners, who had to be informed of the unexpected service cessation. One professional reflected, *“It is frustrating not to be able to refer patients to OASIS-BAR. We had quickly started to consider it as a valuable option in our network.”*.

## 4 Discussion

This pilot study supports the feasibility and acceptability of implementing a standalone specialised service for BD prevention within the NHS in a socially deprived and ethnically diverse inner-city borough with a high incidence of severe mental disorders.

As the first NHS-funded service dedicated to identifying and supporting individuals meeting SIBARS criteria, OASIS-BAR represents a significant milestone in preventive psychiatry.

By addressing feasibility and acceptability, this study offers real-world insights into how BD prevention can be effectively integrated within existing early intervention networks, informing future service design, outreach, and care delivery.

Despite the financial constraints and the reduced staffing, the successful setup of OASIS-BAR demonstrates the feasibility of a BD preventive service grounded in co-production with EbE, multidisciplinary collaboration and specialist knowledge and training.

The dissemination of validated assessment tools, such as the SIBARS, and the increased accessibility of dedicated training programmes can facilitate the wider implementation of BD preventive services despite financial constraints (48).

Bipolar disorders impose a substantial societal and economic burden, with an estimated mean annual cost of approximately £12, 617 per patient in the UK (49). This burden highlights the need to address the outcomes that contribute to these costs, including loss of productivity, disability, relapse, and hospitalisation. Crucially, individuals meeting at-risk criteria are already help-seeking and present with significant functional impairment and reduced quality of life, indicating that early intervention services can address not only illness risk and long-term outcomes but also substantial current clinical needs (50, 51). While a formal cost-effectiveness evaluation was beyond the scope of this feasibility pilot, the OASIS-BAR experience provides preliminary evidence that a dedicated preventive service for individuals at risk of bipolar disorder can be implemented within existing NHS early intervention infrastructure with relatively limited additional resources. In relation to its outreach and referral pathways performance, OASIS-BAR received referrals from a range of sources, reflecting its rapid integration within the broader SLaM care network. The effective collaboration with key referrers, supported by OASIS-BAR’s standalone configuration (52, 53), enabled targeted outreach and timely referral of individuals at risk. Although direct comparisons are limited by differences in target population, diagnostic focus and service maturity, the referral yield was broadly consistent with the early implementation phase of OASIS services for CHR-P individuals, which received 180 referrals over 30 months and identified 58 CHR-P cases (54). The collaboration with CAMHS particularly underscores the importance of transitional services in facilitating early detection during adolescence and in promoting continuity of care for young individuals.

The effective collaboration with other NHS services also contributed to the brief waiting time between referral and assessment in OASIS-BAR. Rapid access to preventive services is crucial, as it has been shown to impact clinical and functional outcomes. A study of 8, 949 service users of NHS early intervention for psychoses services found that longer waiting times were significantly associated with a deterioration in clinical and functional outcomes (55). Evidence on CHR-P individuals suggests that longer duration of untreated prodromal symptoms correlates with poorer long-term functional outcomes and a decreased likelihood of remission (56, 57). In our study, the mean waiting time from referral to a complete assessment was 22.4 days. The Royal College of Psychiatrists’ Early Intervention in Psychosis Network (EIPN) quality standards for an individual with a suspected at-risk mental state (58) require that specialist assessment of adults with a suspected ARMS should be initiated within two weeks of receipt of referral. At OASIS-BAR, the complete diagnostic and clinical formulation was achieved in a mean of 22.4 days, i.e., a more demanding endpoint than assessment initiation and well within the extended-assessment window foreseen by the same standard, indicating a timely assessment pathway in real-world practice.OASIS-BAR achieved optimal detection rates, suggesting that referrals carried a high pretest risk, an essential factor both for the predictive validity of current risk assessment tools (59) as well as for efficient resource allocation to individuals most in need of specialised care (60, 61). This likely reflects the effectiveness of OASIS-BAR’s targeted outreach and close collaboration with other NHS services (59–61), which ensured that referrals were filtered by professional figures familiar with OASIS-BAR, enriching the sample with individuals more likely to meet SIBARS criteria.

In relation to OASIS-BAR’s service provision performance, the range and intensity of delivered interventions demonstrate that multidisciplinary preventive care tailored to the diverse needs of individuals meeting SIBARS criteria can be feasibly implemented even with limited staffing.

In relation to acceptability, OASIS-BAR services achieved sustained engagement from their users; regarding the mean length of stay, it should be noted that most discharges (9/10) happened in the same period (October 2024) through planned discharge arrangements due to the interruption of the service. Professional stakeholders particularly stressed the clinical value of OASIS-BAR as a new complementary resource; service users valued its specialised support in managing their difficulties. In summary, OASIS-BAR demonstrates that specialised, co-designed preventive services for individuals meeting SIBARS criteria are both feasible and acceptable within the NHS, offering a scalable model to enhance early detection and improve outcomes.

### 4.1 Limitations

The findings of the present research report have some limitations. First, because OASIS-BAR represents a novel service model in an emerging field, dedicated guidelines, established benchmarks, and comparable standalone BAR services are currently lacking. Therefore, feasibility and acceptability indicators, including waiting times, recruitment, engagement and retention rates, should be interpreted as preliminary implementation outcomes rather than as evidence of superiority over existing care pathways. Secondly, the short programme duration limited the gathering of longitudinal data on service effectiveness. However, this was beyond the scope of the present report, which focused on the feasibility and acceptability of its pilot implementation. In relation to acceptability, the small sample size precluded quantitative analyses, warranting further data for a more systematic evaluation of acceptability among service users and stakeholders. Moreover, the feedback was gathered on a voluntary basis as part of a service audit rather than through systematic qualitative methodology, which may introduce selection bias. Other limitations stem from the limited financial resources, which hindered staff recruitment, provision of care, and led to the premature interruption of the service. These factors may have confounded both feasibility and acceptability outcomes, reflecting the broader issue of the underfunding of mental health services (3, 62–64) and the difficulty of testing innovative services under financial instability (52).

Finally, the service was piloted in a socially deprived, ethnically diverse inner-city borough characterised by a high incidence of severe mental disorders burdening mental health services. At the same time, the presence of a well-established early intervention network within SLaM may have facilitated implementation and referral pathways. These factors may limit the generalisability of the findings, particularly to low- and middle-income countries.

### 4.2 Future directions

The implementation of dedicated preventive services represents an important step toward generating further empirical evidence on SIBARS constructs, their prognostic value, and on the clinical characterization and detection of BD vulnerability (65). The OASIS-CHR-P services stand out as a leading example, with over 5, 922 citations for contributions to CHR-P research, including on prediction models, neuroimaging, digital mental health, and pharmacological and psychosocial interventions (31). By facilitating larger longitudinal studies and clinical trials, dedicated services can contribute to strengthening the evidence base on the efficacy of BD preventive interventions, which at the moment remains limited but promising (66–68). Reflecting this limited evidence base, no intervention protocol has yet been validated specifically for bipolar at-risk states; accordingly, OASIS-BAR delivered need-based interventions, in line with other early detection services in England (52). Early feasibility work, such as the Bipolar At Risk Trial, has demonstrated that targeted psychological interventions for BAR individuals are feasible, acceptable, and show promising signals of therapeutic benefit (69). The intervention is currently being evaluated in a multicentre randomized controlled trial (the Bipolar At Risk Trial II, BART-II), designed to test the efficacy of a CBT intervention targeting maladaptive appraisal processes to reduce distressing mood swings (70). Several other trials are currently ongoing, underscoring the increasing momentum in this area (70–73).

The dissemination of validated risk-assessment tools, such as the SIBARS (9, 27), is crucial for clinical and research advancement in this field. A key priority would be to promote accessible training programmes for these instruments (e.g. the Maudsley Learning “SIBARS Practitioner Training Course”, https://maudsleylearning.com/courses/structured-interview-for-bipolar-at-risk-states-sibars/) (48); adequate financial support for staff training should be secured through organisational planning and policy-level decisions (65). A key future step is the adoption of digital tools to enhance the real-world penetration of outreach and clinical monitoring strategies (74–76). Youth-friendly digital platforms have been developed to promote help-seeking behaviour in individuals not reached by conventional recruitment (e.g., the OASIS Me and My Mind website, https://www.meandmymind.nhs.uk and the Instagram account @meandmymindoasis) (77). This would be particularly crucial to enhance the detection of younger individuals, closer to the typical age of onset of bipolar disorder, and of groups under-represented in conventional referral pathways, including some minoritised ethnic communities, thereby promoting earlier and more equitable access to preventive care. Digital tools have been used to support risk screening and prediction models (75, 78, 79). In addition, these resources can help raise awareness, reduce stigma, and inform service design based on the needs and preferences of young people (31). Future research contributions will need to maintain the focus on these and other key ethical challenges in early intervention, including communicating risk conditions in an age-sensitive and hope-promoting way (80, 81). Future implementations should also monitor referral equity across ethnic and socioeconomic groups, as the small number of OASIS-BAR referrals precluded reliable conclusions on representativeness. We hope that the present contribution may support the implementation of co-designed, youth-friendly, person-centred, specialized services for individuals at risk of BD.

## Data Availability

The data analyzed in this study is subject to the following licenses/restrictions: The anonymized data that support the findings of this study are available from the corresponding author upon reasonable request. Requests to access these datasets should be directed to antonio.melillo@kcl.ac.uk.
